# A Case of Mirror-Image Crossed Thalamic Aphasia With Jargon Agraphia

**DOI:** 10.7759/cureus.60637

**Published:** 2024-05-19

**Authors:** Nobuhiro Takahashi, Mimpei Kawamura, Mamiko Sato, Yasutaka Kobayashi

**Affiliations:** 1 Department of Rehabilitation, Fukui Health Science University, Fukui, JPN; 2 Department of Medical Welfare, Kyoto Koka Women's University, Kyoto, JPN

**Keywords:** motor memory, jargon agraphia, thalamic infarction, thalamic aphasia, crossed aphasia

## Abstract

In right-handed individuals, aphasia resulting from right hemisphere damage is termed crossed aphasia and has a very low occurrence rate. Additionally, aphasia due to thalamic lesions often involves hemorrhage, with infarction cases less frequently reported. We present the case of an 81-year-old right-handed female who developed aphasia due to a right thalamic infarction. She exhibited characteristics typical of thalamic aphasia observed in left thalamic lesions. Furthermore, jargon agraphia manifested during writing tasks. This may suggest disinhibition of the left hemisphere writing motor memory by the right hemisphere language function.

## Introduction

Thalamic aphasia is a clinical syndrome of acquired language disorder typically resulting from lesions in the cortical-subcortical language network of the left hemisphere [1,2]. The estimated incidence of aphasia following a left thalamic stroke ranges between 12% and 80%, making it common [3]. Conversely, when aphasia occurs owing to damage to the right hemisphere in right-handed individuals, it is termed crossed aphasia. The occurrence rate of crossed aphasia is less than 3% among patients with right hemispheric damage, making it rarely reported [4]. Therefore, the emergence of aphasia symptoms following right thalamic damage is uncommon. Additionally, reports of aphasia due to thalamic lesions are more commonly associated with brain hemorrhages, which often damage tissues outside the thalamus [5]. In contrast, cerebral infarctions do not affect the surrounding tissues, making them important for understanding the function of the thalamus itself [5]. Here, we report a rare case of aphasia following right thalamic infarction in a right-handed individual. Furthermore, we analyze the interesting findings observed during the patient's writing task.

## Case presentation

**Figure 1:**
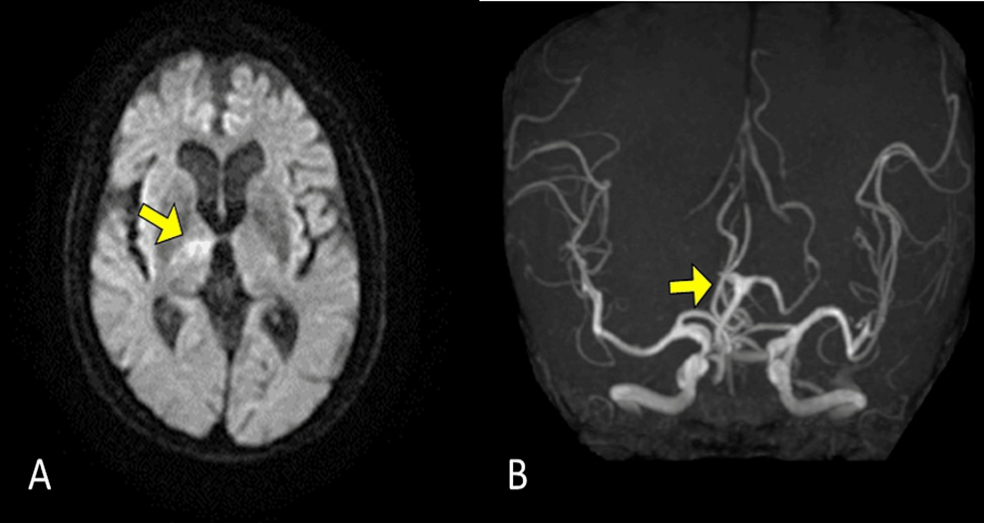
Brain imaging at the onset of cerebral infarction (A) High signal area was observed in the right anterior thalamus on diffusion-weighted imaging. (B) Magnetic resonance angiography did not depict the right posterior cerebral artery P1 segment.

An 81-year-old woman was admitted to the department of orthopedic surgery for left shoulder rotator cuff repair. The patient collapsed during postoperative self-training in the rehabilitation room. Head magnetic resonance imaging with diffusion-weighted imaging revealed a high-signal area in the right thalamus. Magnetic resonance angiography showed an infarction in the right posterior cerebral artery (PCA) P1 segment (Figure 1). Consequently, the patient was transferred to the rehabilitation department. Thrombolytic therapy with tissue plasminogen activator was administered three hours after disease onset, with a 2.6 mL intravenous bolus followed by a continuous infusion at a rate of 23 mL per hour; however, reperfusion of the right PCA was not achieved. Anticoagulation therapy and rehabilitation for poststroke sequelae were initiated the following day. Neurological examination at the start of rehabilitation revealed mild left hemiparesis and decreased sensation in the upper and lower left limbs. Higher levels of cognitive dysfunction, including left-sided neglect and aphasia, were observed. The patient had no history of psychiatric or neurological disorders. The patient scored 28 of 30 points on the Revised Hasegawa Dementia Scale (HDS-R) during admission to the orthopedic surgery department [6]. The HDS-R is commonly used in Japan alongside the Mini-Mental State Examination and has proven useful for detecting dementia [7]. The total score on the HDS-R is 30 points, and a score of 20 or less indicates a high likelihood of dementia; however, there was no evident decline in cognitive function in this case. The Flinders Handedness Survey [8] score was a perfect +10, indicating strong right-handedness. No left-handed or ambidextrous relatives were identified.

**Figure 2:**
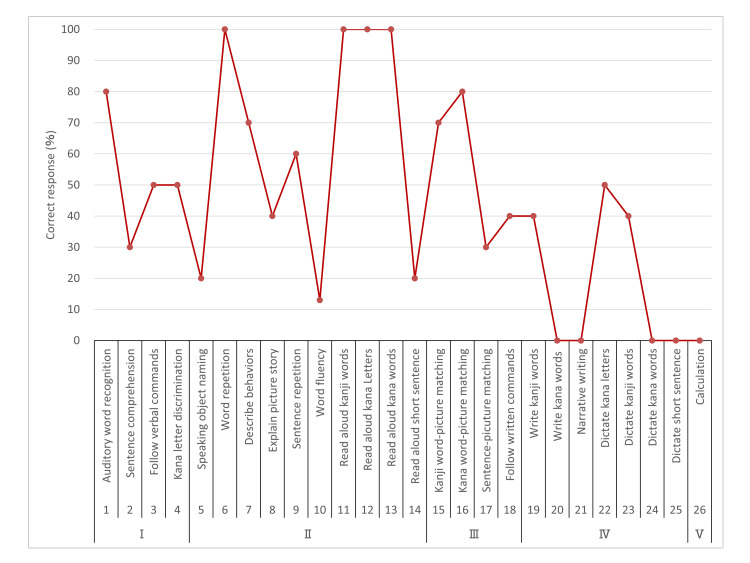
Profile of the Standard Language Test of Aphasia I - auditory comprehension; II - speaking; III - reading comprehension; IV - writing; V - calculation

**Figure 3:**
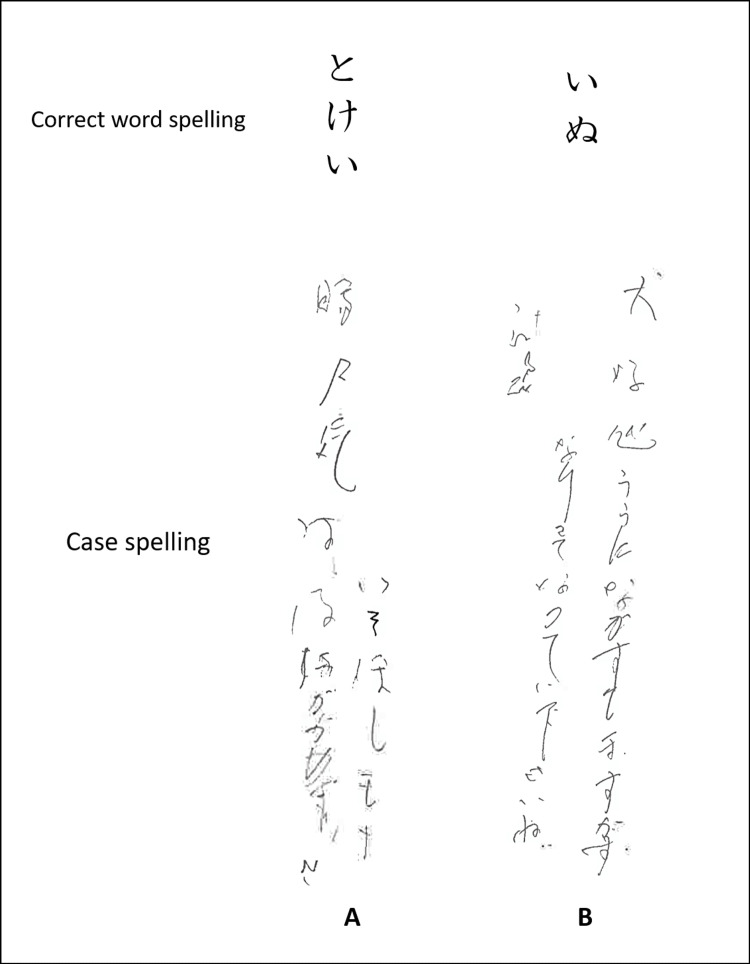
Jargon agraphia observed in the dictate kana words test of the Standard Language Test of Aphasia The patient wrote meaningless character strings without hesitation. (A) Japanese characters representing "clock"; (B) Japanese characters representing "dog"

Neuropsychological assessment was conducted during the second week after onset. A comprehensive evaluation of aphasia was performed using the Standard Language Test of Aphasia [9] (Figure 2). In the auditory and reading comprehension sections, errors in picture-pointing owing to left-sided neglect and attention deficits were frequently observed. However, comprehension at the everyday conversation level was generally preserved. Spontaneous speech was limited, and voice volume decreased; however, there was no apraxia of speech or phonological paraphasia errors. Word repetition and reading of words aloud remained intact. However, naming was impaired, with word-finding difficulty and semantic paraphasia (such as calling a dog "cat", a plane "ship", or a goldfish "deer") observed. Writing was particularly challenging, and jargon agraphia was noted (Figure 3). While accurate copying of characters was possible, there were no errors in the stroke order. The Visual Perception Test for Agnosia [10], including line cancellation, line bisection, and picture drawing, revealed left-sided neglect. The Frontal Assessment Battery (FAB) [11] scored 4 out of 18 points, with continuous tapping observed until inhibition in the "GO/NO-GO" task.

## Discussion

This case had no history of psychiatric or neurological disorders. Despite being relatively older, the patient scored 28 points on the Hasegawa Dementia Scale-Revised before stroke onset, indicating no cognitive impairment, including language function. Despite being right-handed, the patient exhibited aphasia owing to a localized stroke in the right thalamus. Mariën et al. [12] listed five criteria for crossed aphasia as follows: 1) evidence of aphasia on language examination, 2) lesion limited to the right hemisphere, 3) no childhood brain injury, 4) right-handedness, and 5) no family history of left-handedness. This case met all these criteria, suggesting crossed aphasia. Alexander et al. [13] classified crossed aphasia into two types: mirror image-crossed and anomalously crossed aphasia. In mirror image-crossed aphasia, the relationship between the lesion site and aphasia symptoms is similar to aphasia caused by left hemisphere damage. For example, motor aphasia occurs because of frontal lobe damage, whereas sensory aphasia occurs because of temporal lobe damage. Anomalous crossed aphasia refers to cases in which the features of aphasia do not correspond to the lesion site in the left hemisphere. Although various types of aphasia can occur with left thalamic damage, De Witte et al. [14] described six typical features of thalamic aphasia: reduced spontaneous speech or lack of language initiation; decreased volume and/or mild dysarthria; moderate-to-severe naming impairment characterized by semantic paraphasia, neologism, and perseveration; normal or mild repetition impairment; normal or mild language comprehension impairment; and speech fluency. The patient exhibited reduced spontaneous speech and volume. While naming was difficult owing to word-finding difficulties and semantic paraphasia, word repetition at the single-word level was intact. Language comprehension in everyday conversations was generally good. Five of the six features of thalamic aphasia proposed by De Witte et al. were present in this case, suggesting thalamic aphasia due to left thalamic damage and, thus, classified as mirror image-crossed aphasia.

In this case, there was a reduction in spontaneous speech, and the speech was non-fluent. However, in the writing task, the patient promptly spelled many incorrect characters without hesitation. The discrepancy in fluency between the speech and writing tasks is intriguing. Yokoyama et al. [15] discussed jargon agraphia in crossed aphasia, suggesting that the motor memory for writing is embedded in the left hemisphere for individuals with the habit of writing with the right hand. They proposed that this motor memory, when released from the inhibition by the language function of the right hemisphere, autonomously drives the writing motion of the right hand (free-running hypothesis). Subsequent support for the free-running hypothesis has been observed in Japan [16-20]. In this case, the patient was originally right-handed and wrote with the right hand, suggesting the presence of a motor memory for writing in the left hemisphere. The ability to write characters without errors in stroke order suggests that motor memory for writing was not impaired. Additionally, the patient exhibited aphasia due to right thalamic infarction, indicating the presence of language function in the right hemisphere. Furthermore, in accordance with the aforementioned hypothesis, Sato et al. [18] suggested that intentional continuous movement control impairment contributes to the development of jargon agraphia. From the findings of the FAB in this case, it appears that there was an impairment in the intentional continuous movement control of the upper limbs. Based on these factors, it is believed that, in this case, the motor memory for writing in the left hemisphere was released from inhibition by the language functions of the right hemisphere and was further compounded by intentional, continuous motor control impairment, resulting in the emergence of jargon agraphia.

## Conclusions

This was a rare case of a right-handed individual developing aphasia due to a right thalamic infarction. The characteristics of the aphasia resembled those of thalamic aphasia, which is typically observed in left thalamic lesions. Furthermore, jargon agraphia emerged during the writing tasks. This patient exhibited features of crossed aphasia and was thought to involve jargon agraphia related to the disinhibition of motor memory for writing. Conducting large-scale studies on such rare cases is unrealistic because of the difficulty in recruiting subjects. The accumulation of such case reports is expected to contribute to a better understanding of the mechanisms underlying the condition and its treatment.
